# Simple Evaluation of Clinical Situation and Subtypes of Pediatric Hemophagocytic Lymphohistiocytosis by Cytokine Patterns

**DOI:** 10.3389/fimmu.2022.850443

**Published:** 2022-02-28

**Authors:** Xiao-Jun Xu, Ze-Bin Luo, Hua Song, Wei-Qun Xu, Jan-Inge Henter, Ning Zhao, Meng-Hui Wu, Yong-Min Tang

**Affiliations:** ^1^Division/Center of Pediatric Hematology-Oncology, the Children’s Hospital of Zhejiang University School of Medicine, Hangzhou, China; ^2^The Pediatric Leukemia Diagnostic and Therapeutic Technology Research Center of Zhejiang Province, National Clinical Research Center for Child Health, Hangzhou, China; ^3^Childhood Cancer Research Unit, Department of Women’s and Children’s Health, Karolinska Institute, Stockholm, Sweden; ^4^Pediatric Oncology, Theme of Children’s Health, Karolinska University Hospital, Stockholm, Sweden

**Keywords:** hemophagocytic lymphohistiocytosis, cytokines, interleukin-10, interferon-gamma, prognosis

## Abstract

**Background:**

Hemophagocytic lymphohistiocytosis (HLH) is a rapidly fatal disease caused by immune dysregulation. Early initiation of treatment is imperative for saving lives. However, a laboratory approach that could be used to quickly evaluate the HLH subtype and clinical situation is lacking. Our previous studies indicated that cytokines such as interferon (IFN)-γ and interleukin (IL)-10 were helpful for the early diagnosis of HLH and were associated with disease severity. The purpose of this study is to clarify the different cytokine patterns of various subtypes of pediatric HLH and to investigate the role of cytokines in a simple evaluation of disease feature.

**Patients and Methods:**

We enrolled 256 pediatric patients with newly diagnosed HLH. The clinical features and laboratory findings were collected and compared among different subtypes of HLH. A model integrating cytokines was established to stratify HLH patients into different clinical groups.

**Results:**

Twenty-seven patients were diagnosed with primary HLH (pHLH), 179 with EBV-HLH, and 50 with other causes. The IL-6, IL-10, and IFN-γ levels and the ratios of IL-10 to IFN-γ were different among EBV-HLH, other infection-associated HLH, malignancy-associated HLH, familial HLH, and X-linked lymphoproliferative disease. Patients with the ratio of IL-10 to IFN-γ >1.33 and the concentration of IFN-γ ≤225 pg/ml were considered to have pHLH, with a sensitivity of 73% and a specificity of 84%. A four-quadrant model based on the two cutoff values was established to stratify the patients into different clinical situations. The HLH subtypes, cytokine levels, treatment regimens, treatment response, and outcomes were different among the four quadrants, with the 8-week mortality from 2.9 ± 2.9% to 21.4 ± 5.5% and the 5-year overall survival from 93.9 ± 4.2% to 52.6 ± 7.1%.

**Conclusions:**

Different subtypes of HLH present distinct cytokine patterns. IFN-γ and the ratio of IL-10 to IFN-γ are helpful tools to differentiate HLH subtypes. A four-quadrant model based on these two parameters is a useful tool for a simple evaluation of the HLH situation.

## Introduction

Hemophagocytic lymphohistiocytosis (HLH) is a life-threatening entity which presents as multiorgan dysfunction caused by severe hyperinflammation. Overactivated cytotoxic T cells and macrophages and overwhelming cytokine storm contribute to the fatal outcome (1). As hypercytokinemia plays a key role in HLH, we have conducted serum Th1/Th2 cytokine determination by flow cytometry in pediatric patients with HLH since 2005 and reported that the cytokine pattern with significantly increased levels of interferon (IFN)-γ and interleukin (IL)-10 combined with a slightly increased IL-6 level was highly specific for childhood HLH (2). The sensitivity and specificity were both higher than 90% for HLH diagnosis among febrile patients in the pediatric hematology-oncology department when IFN-γ was higher than 75 pg/ml and IL-10 was higher than 60 pg/ml (3). This cytokine pattern has been recommended for the differential diagnosis between secondary HLH and infection (4). Moreover, cytokines such as IFN-γ and IL-10 were closely associated with the severity of organ damage and outcome, and they have been adopted as important biomarkers for risk stratification and treatment option (5, 6). However, as more and more cases of HLH were accumulated, we found that not all HLH patients presented the above cytokine pattern. For example, patients with X-linked lymphoproliferative disease (XLP) and STAT1 gain-of-function mutation presented low levels of IL-10 and IFN-γ (7, 8). A recent report also showed that the levels of IL-6, IL-10, and IFN-γ were different among HLHs driven by different underlying diseases (9). Thus, it is necessary to clarify whether different subtypes of HLH present distinct cytokine patterns to improve the diagnostic accuracy of this test.

Because of the rapid progression of HLH, early initiation of treatment is imperative in order to save lives. A decision on whether or not to use elements of the HLH-94 protocol, including etoposide, is often required when or even before an HLH diagnosis is made (10). However, as the treatment strategies are not the same in different subtypes of HLH, it is a big challenge for clinicians to choose feasible regimens for HLH patients when the underlying causes were not certain. As the cytokine patterns and levels may provide rapid (<5 h after the blood sample taken) information on HLH diagnosis, severity, or even subtypes, it could be a useful evaluation tool when quick decision is required for initial HLH treatment.

Herein, we investigated the clinical data of pediatric patients with HLH initially diagnosed in our institution between January 2010 and December 2020, aiming to investigate the cytokine patterns of different subtypes of HLH and the role of cytokines in the evaluation of the disease feature.

## Patients and Methods

### Patients

This study collected clinical data of pediatric patients with newly diagnosed HLH at the Children’s Hospital of Zhejiang University School of Medicine from January 2010 through December 2020. During the 11 years, a total of 256 patients were enrolled. The diagnosis of HLH was made according to the HLH-2004 protocol (11). Data including the patients’ demographic characteristics, clinical and laboratory findings at diagnosis, treatment regimens, and outcomes were collected for analysis. Patient follow-up was carried out by either outpatient visits and/or individual phone calls. The last follow-up time for this study was March 1, 2021, with a median follow-up time of 44 months.

### Determination of Cytokines

The cytokines including IL-2, IL-4, IL-6, IL-10, tumor necrotic factor (TNF)-α, and IFN-γ were quantitatively measured. The cytokine concentrations at the time of diagnosis of HLH were used for analysis in this study. Determination of the above cytokines was performed by flow cytometry with a commercially available cytometric bead array Human Th1/Th2 Cytokine Kit II, as described previously (2). The lower and upper limits of detection were 1.0 and 5,000 pg/ml for each cytokine. For values higher than 5,000 pg/ml, 5,000 pg/ml was used in the statistical analysis.

### Definition of HLH Subtypes

The Sanger method was used for the HLH-related gene sequencing which included *PRF1*, *UNC13D*, *STX11*, *STXBP2*, *LYST*, *AP3B1*, *RAB27A*, *SH2D1A*, *BIRC4*, *CD27*, *ITK*, and *MAGT1* before January 2017, and whole exome sequencing was used to determine variants in HLH and primary immunodeficiency-related genes afterward. Familial HLH (FHL) was defined as HLH caused by genetic defects in *PRF1*, *UNC13D*, *STX11*, or *STXBP2* which results in defective lymphocyte granule-mediated cytotoxicity of NK and T cells. Primary HLH (pHLH) was defined as presence of genetic inborn errors of immunity with HLH as a main feature of the disease, including FHL and HLH caused by *RAB27A*, *LYST*, *AP3B1*, *SH2D1A*, and *BIRC4* variants (1, 12). Epstein-Barr virus-associated HLH (EBV-HLH) was defined as secondary HLH (sHLH) caused by acute EBV infection which was proven by serological results for antibodies or real-time polymerase chain reaction for EBV DNA, without the presence of the pathogenic HLH-associated genetic variants or malignancies.

### Statistical Analysis

The comparisons of demographic features and laboratory findings in different groups were performed using the Mann–Whitney U test (two groups) or Kruskal–Wallis H test (three or more groups). The performance of biomarkers to differentiate HLH subtypes was assessed with receiver operating characteristic (ROC) curves. The optimal cutoff values were selected by Youden index. Survival curves were performed by the Kaplan–Meier method and compared using the log-rank test. The survival interval was censored at the 5th year in the curves. All statistical analyses were performed using SPSS version 20.0 and GraphPad Prism version 9.3.0. p < 0.05 was considered to be statistically significant.

## Results

### Patients’ Characteristics

Altogether, 256 pediatric patients with HLH were enrolled in this study, with a median age of 2.6 years (range: 3 days to 14.2 years). According to the genetic variant results and underlying causes, this cohort consisted of 27 patients with pHLH (26 by genetic variants and 1 by family history), 179 patients with EBV-HLH (including 39 patients without receiving genetic sequencing), and 50 patients with other causes (20 with infection other than EBV, 2 with systemic-onset juvenile idiopathic arthritis, 1 with Kawasaki disease, 8 with lymphoma/leukemia, 3 with chronic active EBV(CAEBV), and 16 with undetermined underlying causes). Excluding patients with undetermined causes, altogether 213 patients were considered as sHLH. Sixteen patients with undetermined causes were included when analyzing the characteristics of the whole cohort, while they were excluded when comparing different features among patients with different subtypes of HLH. The demographic characteristics and clinical features of patients with FHL, XLP, EBV-HLH, infection-associated HLH triggered by pathogens other than EBV (I-HLH), and malignancy-associated HLH (M-HLH) are listed in **Table 1**. The median EBV load was 1.45 × 10^5^ copies/ml in the sera in patients with EBV-HLH, ranging from 500 copies/ml to 9.02 × 10^6^ copies/ml. As to the treatment regimens, 69 patients received dexamethasone (DXM) only, 19 received DXM and cyclosporin A (CSA), 150 received etoposide-containing regimens (HLH-94/2004), and 18 received other regimens. Only 13 patients underwent hematopoietic stem cell transplantation. The 5-year overall survival of this cohort was 70.0 ± 2.9%.

**Table 1:** Comparison of clinical characteristics and laboratory findings among patients with different subtypes of HLH (median and range).

Parameters	Total	Primary HLH		Secondary HLH			p-value^a^
		FHL	XLP	EBV-HLH	I-HLH	M-HLH	
Median age (year)	2.60 (0.01–14.20)	0.7 (0.1–6.3)	3.2 (0.9–7.7)	2.7 (0.2–14.0)	1.4 (0.1–13.5)	11.7 (2.3–14.2)	**<0.001**
Male to female	133/123	12/6	9/0	79/100	12/8	6/2	**0.003**
Neutrophils (×10^9^/L)	0.67 (0.03–10.07)	0.6 (0.04–9.8)	1.4 (0.4–3.4)	0.6 (0.01–10.1)	1.4 (0.08–11.9)	0.9 (0.15–3.9)	**0.009**
Hemoglobin (g/L)	91 (45–136)	83 (52–111)	90 (59–121)	93 (45–136)	89 (62–117)	96 (58–134)	0.103
Platelet (×10^9^/L)	50 (2–360)	35 (7–266)	138 (6–328)	48 (2–360)	52 (13–410)	35 (1–127)	0.144
ALT (U/L)	166 (13–5910)	68 (15–1665)	305 (27–560)	141 (13–1765)	144 (23–5910)	134 (40–520)	0.234
AST (U/L)	298 (26–24310)	122 (33–9897)	439 (37–1401)	314 (30–4072)	291 (26–24310)	334 (76–718)	0.131
LDH (U/L)	1,285 (262–16,200)	530 (287–13,399)	809 (415–1137)	1,405 (262–12,103)	1,636 (263–16,200)	1,103 (705–1421)	**<0.001**
Albumin (g/L)	29.9 (16.9–53.7)	31.5 (24.6–38.9)	25.8 (18.7–32.3)	30.1 (17.1–53.7)	28.9 (16.9–35.1)	32.2 (22.9–36.2)	0.126
Bilirubin (μmol/L)	20.4 (1.0–132.5)	18.5 (4.3–132.5)	36.8 (4.3–115.6)	19.5 (1.0–131.7)	37.7 (2.6–125.0)	52.2 (9.4–110.0)	0.448
Triglyceride (mmol/L)	2.81 (0.59–21.20)	2.26 (1.06–7.62)	2.31 (1.28–21.20)	2.99 (0.59–20.27)	2.43 (1.63–11.96)	2.49 (1.17–4.33)	0.094
Fibrinogen (g/L)	1.08 (0.10–5.06)	1.24 (0.10–2.88)	1.11 (0.67–1.96)	1.10 (0.22–3.43)	1.07 (0.16–5.06)	1.42 (0.63–2.03)	0.868
D-Dimer (mg/L)	6.58 (0.27–85.40)	3.65 (0.57–25.12)	4.26 (2.13–14.76)	6.73 (0.27–85.40)	5.17 (2.18–24.00)	12.16 (3.95–20.00)	0.252
Urine nitrogen (mmol/L)	3.87 (0.92–31.05)	3.47 (1.46–7.63)	3.15 (2.31–5.05)	3.96 (1.43–31.05)	3.55 (1.15–12.28)	3.53 (2.83–5.21)	0.409
Creatinine (μmol/L)	39.0 (5.0–410.0)	37.0 (21.7–52.0)	30.6 (8.0–56.0)	39.0 (13.3–410.0)	43.0 (5.0–177.0)	50.0 (33.0–63.0)	0.329
Acetylcholinesterase (U/L)	3,002 (891–9559)	4,030 (1829–8168)	3,002 (2518–6421)	4,347 (210–9559)	3,895 (1670–6341)	5,129 (3192–5936)	0.306
Ferritin ≥ 1,500 ng/ml	210/256 (82.0%)	15/18 (83.3%)	6/9 (66.7%)	148/179 (82.7%)	17/20 (85.0%)	7/8 (87.5%)	0.773
Soluble CD25 (pg/mL)	12,325 (425–47,028)	8,204 (3,225–23,119)	14,398 (4,318–24,478)	10,861 (425–47,028)	12,738 (,5768–39,994)	6,296 (5,824–6,768)	0.949

ALT, alanine aminotransferase; AST, aspartate aminotransferase; LDH, lactate dehydrogenase, FHL, familial HLH; XLP, X-linked lymphoproliferative disease; I-HLH, infection-associated HLH triggered by pathogens other than EBV; M-HLH, malignancy-associated HLH.

^a^comparisons among the five groups.

A total of 169 (66.0%) patients underwent gene sequencing, including 82 with Sanger sequencing and 87 with whole exon sequencing. Of all the patients that underwent gene screening, no HLH-related genetic variant was found in 87 patients. Twenty-six of 169 (14.8%) patients were diagnosed with pHLH, including 4 with FHL2, 8 with FHL3, 3 with FHL5, 7 with XLP1, 2 with XLP2, 1 with *LYST* variant, and 1 with the *RAB27A* variant. Homozygous mutation was not found in this cohort. All patients presented either compound heterozygous or hemizygous mutations (**Supplementary Table**).

### Th1/Th2 Cytokine Levels in Pediatric HLH

**Figure 1:**
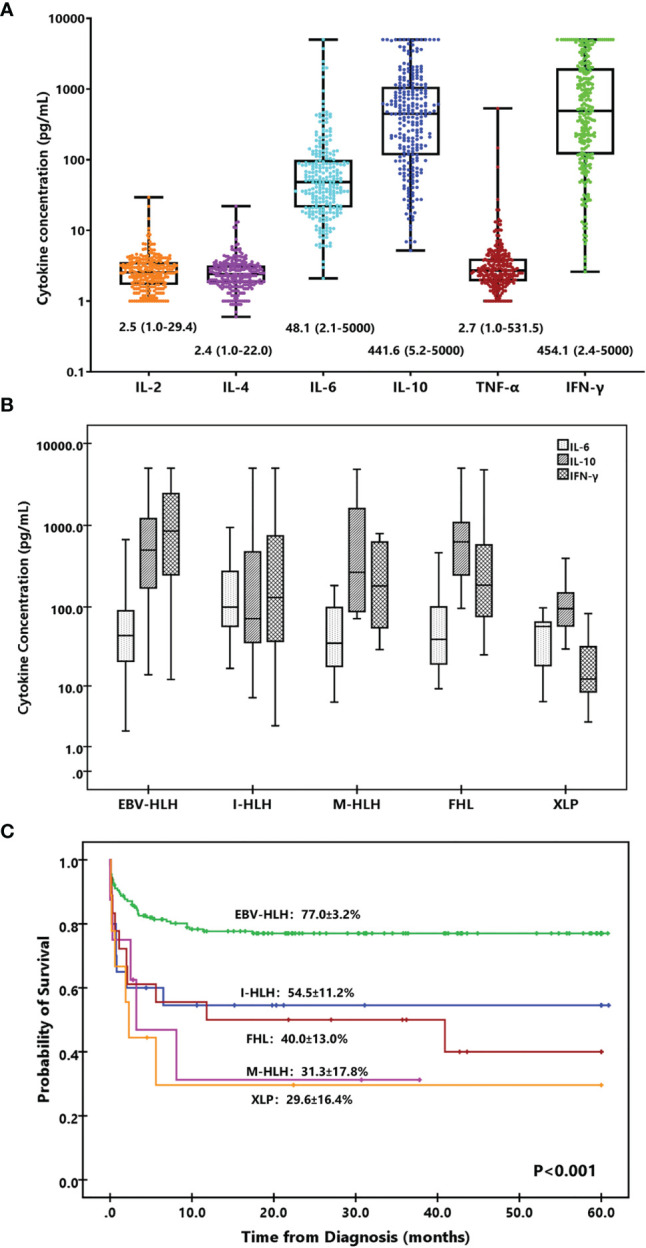
Cytokine levels and cytokine patterns. **(A)** Box-and-whisker plots of IL-2, IL-4, IL-6, IL-10, TNF-α, and IFN-γ levels in HLH. Median levels (lines), 25th to 75th percentiles (box), and minimum to maximum values (whiskers) are shown for each cytokine. The numbers are the median and range of each cytokine. **(B)** Distinct cytokine patterns of different forms of HLH based on IL-6, IL-10, and IFN-γ. Median levels (lines), 25th to 75th percentiles (box), and 5th to 95th percentiles (whiskers) are shown for each cytokine. **(C)** Kaplan–Meier survival curves for 5-year overall survival (OS) of the patients with different subtypes of HLH. I-HLH, infection-associated HLH triggered by pathogens other than EBV; M-HLH, malignancy-associated HLH; FHL, familial HLH; XLP, X-linked lymphoproliferative disease.

Th1/Th2 cytokines including IL-2, IL-4, IL-6, IL-10, TNF-α, and IFN-γ were investigated in the present study. The median concentrations of IL-2, IL-4, and TNF-α were 2.5, 2.4, and 2.7 pg/ml, and those of IL-6, IL-10, and IFN-γ were 48.1, 441.6, and 454.1 pg/ml, respectively (**Figure 1A**). Only 4 (1.6%), 31 (12.1%), and 35 (13.7%) patients presented increased IL-2, IL-4, and TNF-α, while 215 (84.0%), 256 (100%), and 238 (93.0%) patients presented elevated IL-6, IL-10, and IFN-γ, respectively. Thus, only IL-6, IL-10, and IFN-γ were considered valuable for HLH diagnosis and used for further analysis. If we set the HLH cytokine criteria as IL-10 higher than 60 pg/ml and IFN-γ higher than 75 pg/ml based on our previous study, 193 (75.4%) patients fulfilled the criteria, including 14 of 18 (77.7%) with FHL, 2 of 9 (22.2%) with XLP, 152 of 179 (84.9%) with EBV-HLH, and 13 of 28 (46.4%) with sHLH caused by other triggers (*p* < 0.001). The cytokines decreased dramatically after treatment. IL-6, IL-10, and IFN-γ decreased to median levels of 10.9, 35.2, and 26.4 pg/ml in 48–72 h after initial treatment, respectively.

### Cytokine Levels and Patterns in Different Subtypes of HLH

Although most HLH patients presented a cytokine pattern of elevated IL-10 and IFN-γ, the levels and the ratios of these cytokines were different among various forms of HLH. The IL-6 and IL-10 levels were comparable between primary and secondary HLH (median of IL-6, 48.0 vs. 45.8 pg/ml, *p* = 0.902; median of IL-10, 396.2 vs. 459.2 pg/ml, *p* = 0.659), while the IFN-γ level was much lower in pHLH (median, 85.5 vs. 694.9 pg/ml, *p* < 0.001). We further compared with IL-6, IL-10, and IFN-γ concentrations in patients with EBV-HLH, I-HLH, M-HLH, FHL, and XLP. As shown in **Table 2**, patients with I-HLH presented a higher IL-6 level than other forms of HLH. Patients with EBV-HLH, I-HLH, M-HLH, and FHL presented similar levels of IL-10, which was much higher than that in XLP. The IFN-γ level in EBV-HLH was much higher than those in other groups. The IFN-γ concentration was normal or slightly elevated in most XLP patients.

**Table 2:** Cytokine levels and ratios in different forms of HLH.

	IL-6	IL-10	IFN-γ	IL-10/IFN-γ	IL-6/IFN-γ	IL-6/IL-10
Total	48.1	446.3	489.9	0.73	0.85	0.11
(n = 256)	(2.1–5,000)	(5.2–5,000)	(2.6–5,000)	(0.01–124.43)	(0.001–34.45)	(0.01–25.64)
sHLH	45.8	459.2	694.9	0.64	0.08	0.11
(n = 213)	(2.1–5,000)	(5.2–5,000)	(2.6–5,000)	(0.01–20.52)	(0.001–24.85)	(0.01–25.6)
pHLH	48.0	396.2	85.5	2.98	0.36	0.16
(n = 27)	(6.1–462.7)	(11.6–5,000)	(3.0–4,773.5)	(0.27–124.43)	(0.03–34.45)	(0.01–5.56)
sHLH						
EBV-HLH	44.2	492.4	871.1	0.63	0.06	0.09
(n = 179)	(2.1–5,000)	(5.2–5,000)	(4.2–5,000)	(0.01–14.79)	(0.001–6.0)	(0.01–25.64)
I-HLH	100.3	72.4	131.3	1.09	0.78	1.27
(n = 20)	(17.0–942.4)	(6.9–5,000)	(2.6–5,000)	(0.03–11.92)	(0.04–24.85)	(0.02–9.91)
M-HLH	27.1	361.3	181.6	1.88	0.22	0.16
(n = 8)	(6.0–183.7)	(71.9–4,826.9)	(29.5–795.3)	(0.42–20.52)	(0.03–0.60)	(0.01–0.97)
pHLH						
FHL *^a^*	42.0	660.5	198.0	2.42	0.24	0.09
(n = 18)	(9.2–462.7)	(96.0–5,000)	(25.3–4,773.5)	(0.59–14.70)	(0.03–2.38)	(0.01–0.43)
XLP	57.6	95.5	12.4	3.87	2.50	0.70
(n = 9)	(6.1–427.2)	(11.6–920.8)	(3.0–226.5)	(0.27–124.43)	(0.07–34.45)	(0.02–5.56)
p-value *^b^*	0.902	0.659	< 0.001	< 0.001	< 0.001	0.705
p-value *^c^*	0.029	0.002	< 0.001	<0.001	< 0.001	< 0.001

a Including patient with LYST and RAB27A variants.

b Comparisons between sHLH and pHLH.

c Comparisons among EBV-HLH, I-HLH, M-HLH, FHL, and XLP.

sHLH, secondary HLH; pHLH, primary HLH; I-HLH, infection-associated HLH triggered by pathogens other than EBV; M-HLH, malignancy-associated HLH; FHL, familial HLH; XLP, X-linked lymphoproliferative disease.

According to the IL-6, IL-10, and IFN-γ concentrations and their ratios, we investigated the cytokine patterns of different subtypes of HLH. As shown in **Figure 1B**, the pattern of EBV-HLH was significantly elevated IL-10 and IFN-γ with slightly elevated IL-6, and IFN-γ usually higher than IL-10; the pattern of I-HLH was moderately elevated IL-6, IL-10, and IFN-γ; the patterns of M-HLH and FHL were similar, presenting markedly elevated IL-10 and IFN-γ with slightly elevated IL-6, and IFN-γ usually lower than IL-10; the pattern of XLP was moderately elevated IL-6 and IL-10, while IFN-γ was normal or slightly increased. Patients with EBV-HLH presented superior outcome than those with other forms of HLH (**Figure 1C**).

### Distinguishment of pHLH and sHLH by IFN-γ and the Ratio of IL-10 to IFN-γ

**Figure 2:**
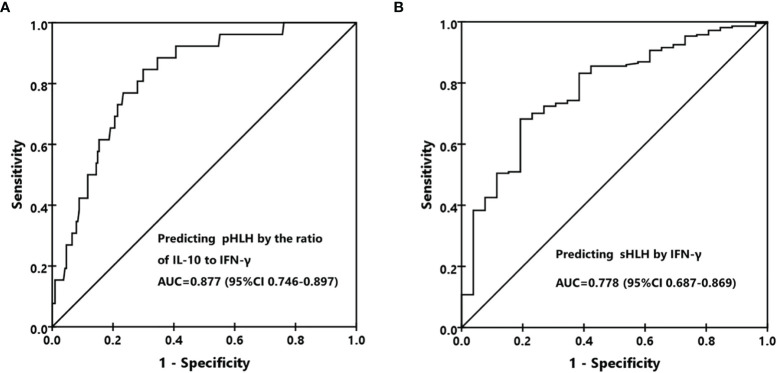
Differentiation of primary HLH (pHLH) and secondary HLH (sHLH). **(A)** The receiver operating characteristic (ROC) curve for predicting pHLH by the ratio of IL-10 to IFN-γ. **(B)** The ROC curve for predicting sHLH by the IFN-γ level.

As different forms of HLH presented distinct cytokine patterns and levels, we then investigated whether different HLH subtypes could be differentiated by cytokine ratios and levels. It was quite common that the IL-10 concentration was higher than that of IFN-γ in patients with pHLH (24/27, 88.9%), while only 36.6% (78/213) of patients with sHLH presented such pattern. Although the ratios of IL-6 to IFN-γ were different between pHLH and sHLH, they were both smaller than 1.0 (**Table 2**). We thus evaluated the performance of the ratio of IL-10 to IFN-γ to differentiate pHLH from sHLH. The results showed that the ratio of IL-10 to IFN-γ could be used as a marker to predict pHLH with an AUC of 0.877(95% CI 0.746-0.897) in ROC analysis (**Figure 2A**). The sensitivity and specificity reached 85.2% and 70.4% at the cutoff value of 1.33. As IFN-γ was much higher in patients with sHLH than in those with pHLH, it could be used as a biomarker to predict sHLH as well. The AUC was 0.778 (95% CI 0.687-0.869) in ROC analysis, with a sensitivity of 70.4% and a specificity of 70.4% at the cutoff value of 225 pg/ml (**Figure 2B**). Whether IFN-γ and the ratio of IL-10 to IFN-γ could be used to differentiate different forms of sHLH was investigated as well. The ratio of IL-10 to IFN-γ could predict M-HLH with an AUC of 0.751 (95% CI, 0.598-0.903). The AUC for IFN-γ to predict EBV-HLH was 0.723 (95% CI, 0.611–0.836).

If we integrated the two biomarkers together to predict pHLH using the ratio of IL-10 to IFN-γ higher than 1.33 and the IFN-γ level less than 225 pg/ml, the specificity, sensitivity, positive predictive value, and negative predictive value were 0.73 (95% CI, 0.54–0.86), 0.84 (0.78–0.88), 0.35 (0.24–0.48), and 0.96 (95% CI, 0.92–0.98), respectively. The positive likelihood ratio, negative likelihood ratio, and diagnostic odds ratio were 4.47 (95% CI, 3.05–6.55), 0.32 (95% CI, 0.17–0.61), and 13.88 (95% CI, 5.43–35.51), respectively.

### Distinct Features of HLH Patients in a Four-Quadrant Model

**Figure 3:**
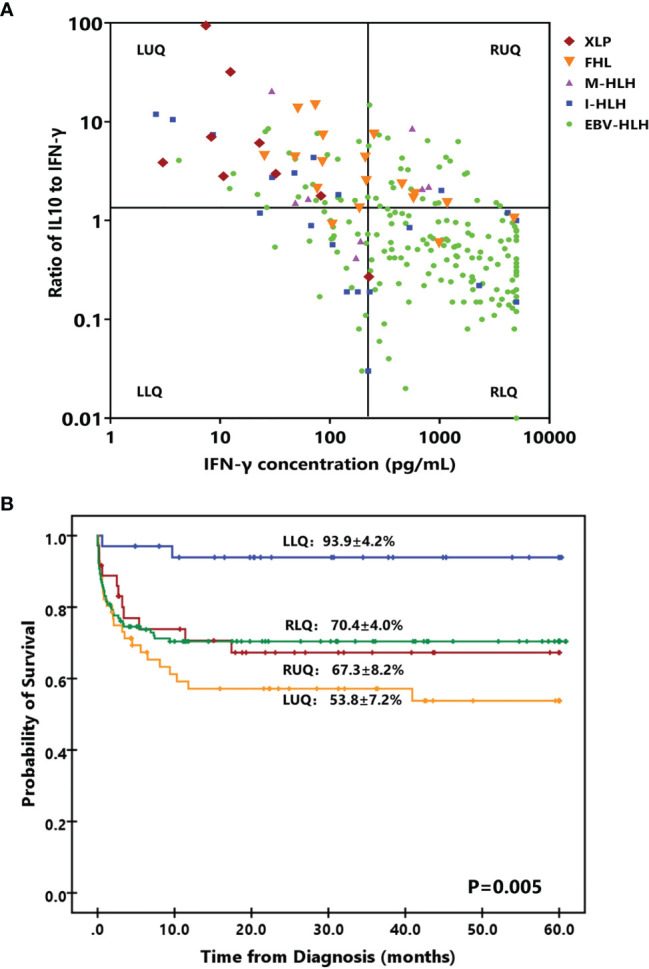
A four-quadrant model to differentiate HLH patients with different features. **(A)** Distribution of patients with EBV-HLH, I-HLH, M-HLH, FHL, and XLP in a four-quadrant diagram based on the ratio of IL-10 to IFN-γ (> 1.33 or ≤ 1.33) and the IFN-γ level (> 225 pg/ml or ≤ 225 pg/ml). **(B)** Kaplan–Meier survival curves for 5-year overall survival (OS) of the patients located in four quadrants. I-HLH, infection-associated HLH triggered by pathogens other than EBV; M-HLH, malignancy-associated HLH; FHL, familial HLH; XLP, X-linked lymphoproliferative disease; LLR, left lower quadrant; LUR, left upper quadrant; RUQ, right upper quadrant; RLQ, right lower quadrant.

To investigate the treatment regimens and outcomes of patients with different cytokine patterns, we established a four-quadrant diagram based on the ratio of IL-10 to IFN-γ (>1.33 or ≤1.33) and the IFN-γ level (>225 or ≤225 pg/ml). As shown in **Figure 3A** and **Table 3**, nearly two-thirds of patients with EBV-HLH were located in the right lower quadrant (RLQ), while most patients with FHL and XLP were located in the left upper quadrant (LUQ). The distribution of I-HLH and M-HLH was scattered. As to the treatment regimens, about half of patients in the left lower quadrant (LLQ) were treated with DXM only without any death, while patients in other quadrants were mainly treated with HLH-94/2004 regimens. The outcome of patients in the four quadrants is shown in **Figure 3B**; patients located in the LLQ presented the best outcome, with the 8-week mortality of 2.9 ± 2.9% and 5-year OS of 93.9 ± 4.2%, while patients in the LUQ presented the poorest outcome, with the 5-year OS of 52.6 ± 7.1%. Although patients located in the RLQ and LUQ presented a similar 8-week mortality, the mortality after 8 weeks was much high for patients in LUQ (33.1 ± 7.8% vs. 10.2 ± 3.1%, *p* = 0.002), mainly due to the persistence or reactivation of disease. A similar tendency was found for patients in RUQ and RLQ (mortality after 8 weeks, 24.2 ± 8.0% vs. 10.2 ± 3.1%, *p* = 0.054).

**Table 3:** Clinical features of HLH patients located in different quadrants in a four-quadrant model.

	Left lower quadrant	Left upper quadrant	Right upper quadrant	Right lower quadrant
**Definition (**IL-10/IFN-γ, IFN-γ**)**	≤ 1.33, ≤ 225	> 1.33, ≤ 225	> 1.33, > 225	≤ 1.33, > 225
**HLH subtypes *^a^***				
EBV-HLH	19 (1, 5.2%)	22 (4, 18.2%)	27 (7, 25.9%)	112 (28, 25.0%)
I-HLH	6 (0, 0.0%)	7 (4, 57.1%)	1 (1, 100%)	6 (4, 66.7%)
M-HLH	2 (0, 0.0%)	3 (3, 100%)	3 (2, 66.7%)	0 (0, 0.0%)
FHL *^d^*	1 (1, 100%)	10 (7, 70.0%)	5 (1, 20.0%)	2 (1, 50.0%)
XLP	0 (0, 0.0%)	8 (4, 50.0%)	0 (0, 0.0%)	1 (1, 100%)
**Treatment regimens *^a^***				
DXM only	18 (0, 0.0%)	17 (4, 23.5%)	7 (1, 14.3%)	27 (8, 29.6%)
DXM/CSA	1 (0, 0.0%)	5 (4, 80.0%)	1 (0, 0%)	12 (5, 41.7%)
HLH-94/2004	11 (2, 18.2%)	28 (13, 46.4%)	25 (8, 32.0%)	87 (21, 24.1%)
Others	4 (0, 0.0%)	7 (3, 42.9%)	3 (2, 66.7%)	4 (4, 100.0%)
**Outcome**				
5-year OS (%) *^b^*	93.9 ± 4.2	52.6 ± 7.1	67.3 ± 8.2	70.4 ± 4.0
8-week mortality (%) *^c^*	2.9 ± 2.9	21.4 ± 5.5	11.2 ± 5.3	21.6 ± 3.6

a Numbers in the bracket are the numbers and percentages of dead patients.

b p = 0.003.

c p = 0.057.

d Including patient with LYST and RAB27A variants.

I-HLH, infection-associated HLH triggered by other pathogens; M-HLH, malignancy-associated HLH; FHL, familial HLH; XLP, X-linked lymphoproliferative disease; DXM, dexamethasone; CSA, cyclosporin A; OS, overall survival.

**Figure 4:**
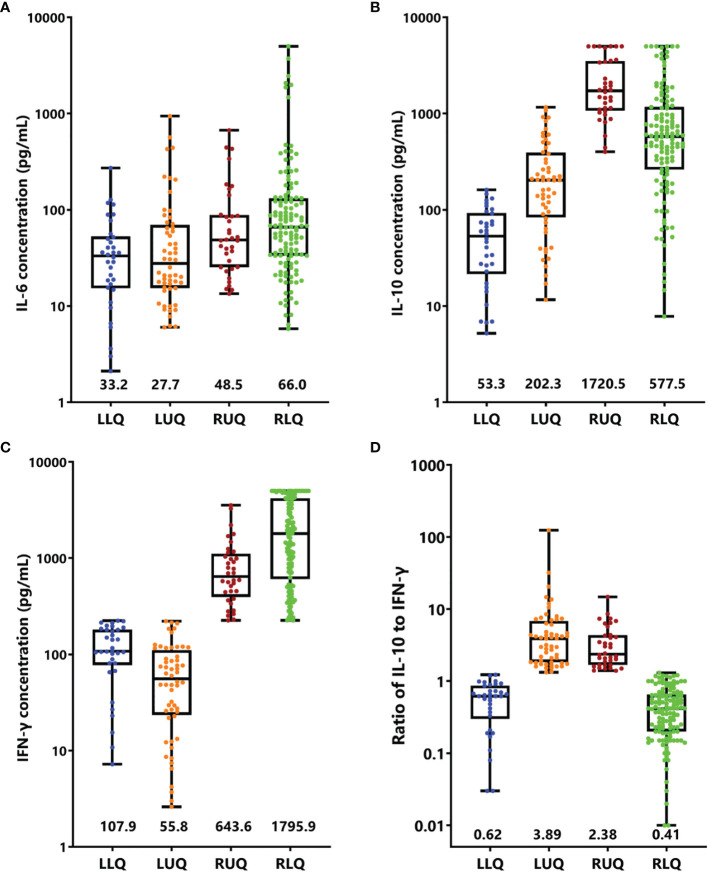
Cytokine levels and ratios in four quadrants. Box-and-whisker plots of IL-6 **(A)**, IL-10 **(B)**, IFN-γ **(C)**, and ratio of IL-10 to IFN-γ **(D)** were shown. Median levels (lines), 25th to 75th percentiles (box), and minimum to maximum values (whiskers) are shown. The numbers in the plots are the median concentration of each cytokine or ratio. LLR, left lower quadrant; LUR, left upper quadrant; RUQ, right upper quadrant; RLQ, right lower quadrant.

The cytokine features of the four quadrants are shown in **Figure 4**; patients in the RUQ presented a much higher level of IL-10 than RLQ, but their IFN-γ level was much lower. The outcomes of patients with EBV-HLH in these two quadrants were similar. Patients in LLQ and LUQ both presented significantly lower IL-10 and IFN-γ levels than those in RUQ and RLQ, but the outcomes of the two populations seemed different even with the same subtype. For patients with sHLH (40 EBV-HLH, 13 I-HLH, and 5 M-HLH totally in the LLQ and LUQ), patients in the LLQ presented a much higher 5-year OS than those in the LUQ (96.0 ± 3.9% vs. 64.1 ± 8.8%, *p* = 0.003), indicating high IL-10 to IFN-γ indicating a poor prognosis for patients with a low IFN-γ level.

As the clinical course of EBV-HLH is a wide range, we thus validated the four-quadrant model in patients with EBV-HLH. There were 19, 22, 26, and 112 patients in the LLQ, LUQ, RUQ, and RLQ, respectively. Patients in the RLQ seemed to have more severe diseases, with higher total bilirubin (*p* = 0.021), alanine aminotransferase (*p* = 0.013), aspartate aminotransferase (*p* < 0.001), lactate dehydrogenase (*p* < 0.001), and D-dimer (*p* < 0.001) and lower fibrinogen (*p* = 0.010) than those in other quadrants. The patients treated with HLH-94/2004 accounted for 31.6%, 50.0%, 80.8%, and 73.2%, while those treated with DXM only accounted for 52.6%, 36.4%, 11.5%, and 18.8% in the LLQ, LUQ, RUQ, and RLQ, respectively (*p* = 0.005). The 28-day mortalities were 0%, 4.5 ± 4.4%, 4.0 ± 3.9%, and 17.9 ± 3.6% for EBV-HLH patients in the LLQ, LUQ, RUQ, and RLQ (*p* = 0.034), and the 5-year OS rates were 94.1 ± 5.7%, 81.1 ± 8.6%, 70.4 ± 9.5%, and 74.7 ± 7.1% (*p* = 0.24), respectively.

## Discussion

HLH is a rapidly fatal disease caused by an immune dysregulation characterized by hypercytokinemia and overactivation of T cells and macrophages. Many inflammatory cytokines including IFN-γ, IL-6, IL-10, IL-12, IL-18, TNF-α, and CXCL9 are elevated in patients with HLH, and the cytokine patterns are helpful in differentiating HLH from infections or other inflammatory entities (13–16). The present study shows that different subtypes of HLH present distinct cytokine patterns. The cytokine pattern of EBV-HLH is similar to what we have reported previously while patients with infection-associated HLH caused by other pathogens present higher IL-6 but moderately increased IL-10 and IFN-γ (3). Patients with FHL had a much lower IFN-γ level, although IL-10 levels were comparable with EBV-HLH. Patients with XLP showed only slightly elevated IL-6, IL-10, and IFN-γ levels, while IL-10 was often much higher than IFN-γ. The IFN-γ level and the ratio of IL-10 to IFN-γ were useful tools to distinguish pHLH from sHLH.

There is growing appreciation that the patterns of T cell activation and cytokine profiles differ considerably in different forms of HLH, which suggests possible underlying differences in the pathogenesis of these entities (17). For example, IL-6 is higher in HLH driven by auto-inflammatory disorders and Kawasaki disease than that in pHLH and EBV-HLH (9, 18). However, the role of IL-10 and IFN-γ has not been fully illustrated. IFN-γ is believed to play a key role in the development of HLH, which has been proved by immune-deficient mouse models (19). In FHL, due to the defective cytolysis of T and NK cells, persistent antigen presentation and stimulation lead to sustained activation of CD8+ T cells, which then results in the release of large amounts of IFN-γ (20). In patients with XLP1, *SH2D1A* deficiency results in the impairment of T and NK cell activation and IFN-γ production is limited (21). In EBV-HLH, EBV-infected T and NK cells in addition to B cells are quite common. The LMP1 expression in T cells upregulated the IFN-γ secretion *via* TRAF-NF-κB signals (22). In M-HLH, transformed cells may drive HLH through upregulated production of cytokines and/or sustained presentation of EBV antigens (23). Thus, the different pathogenesis of various forms of HLH contributes to the distinct cytokine patterns.

Due to the acute onset of most HLH patients with early deterioration and rapid death, early death is an important issue in pediatric HLH (24). Immediate evaluation of HLH and its severity is required so that an early treatment can be quickly initiated. Some prognostic models integrating clinical and laboratory parameters presented excellent performance both in adults and in children (25–27). However, the approach that could be used to quickly evaluate the HLH subtype and clinical situation is lacking. As shown in the present study, we developed a four-quadrant model using IFN-γ level and the ratio of IL-10 to IFN-γ, which is helpful to evaluate the patients’ clinical situation. Patients in the LLQ presented low cytokine levels and excellent outcome and may be cured by less intensive therapy such as DXM only. Patients in the LUQ were more likely to be pHLH cases, and the long-term survival was dismal due to high rates of early death and disease reactivation. Thus, intensive monitoring and sequential evaluation were needed for those patients. Patients in the RUQ could be FHL and M-HLH cases while those in the RLQ were mostly EBV-HLH cases. They both presented intermediate risk of death and needed relatively intensive regimens like the HLH-94 protocol (**Table 4**). By this four-quadrant model, HLH patients were stratified into different risk groups, which could be valuable to improve the outcome and to reduce treatment-related toxicity.

**Table 4:** Summary of clinical features of HLH subtypes based on four-quadrant model.

Regions	Definition	Cytokine level	Main subtype	Risk of fatal outcome	Risk of early death	Risk of persistent disease or reactivation	Suggested initial regimens
LLQ	IL-10/IFN-γ ≤ 1.33 and IFN-γ ≤ 225	Low	EBV-HLH, I-HLH	Low	Low	Low	DXM
LUQ	IL-10/IFN-γ > 1.33 and IFN-γ ≤ 225	Low	FHL, XLP, M-HLH	High	High	High	HLH-94
RUQ	IL-10/IFN-γ > 1.33 and IFN-γ > 225	High	FHL, M-HLH	Intermediate	Intermediate	Intermediate	HLH-94
RLQ	IL-10/IFN-γ ≤ 1.33 and IFN-γ > 225	High	EBV-HLH	Intermediate	High	Low	DXM or HLH-94

LLQ, left lower quadrant; LUQ, left upper quadrant; RUQ, right upper quadrant; RLQ, right lower quadrant; I-HLH, infection-associated HLH triggered by pathogens other than EBV; M-HLH, malignancy-associated HLH; FHL, familial HLH; XLP, X-linked lymphoproliferative disease; DXM, dexamethasone.

Although this is a large cohort of pediatric HLH study, there were some limitations. Firstly, due to the resource limit, only part of patients performed sCD25, CD107a, and NK cytotoxicity measurement, and the upper limit of ferritin was 1,500 ng/ml in our institution; thus, we were not able to perform a detailed statistical analysis on these biomarkers. Secondly, this is a retrospective study, with high risk for bias when analyzing treatment and outcome. Thirdly, as this study was performed in a single center, the reproducibility of our data and conclusion warrants further validation in other institutions. Prospective multicenter studies are warranted to confirm the results and conclusions drawn by the present study.

## Data Availability Statement

The raw data supporting the conclusions of this article will be made available by the authors, without undue reservation.

## Ethics Statement

This study was approved by the Ethics Committee of the Children’s Hospital of Zhejiang University School of Medicine (approval no. 2016-IRB-005). Written informed consent to participate in this study was provided by the participants’ legal guardian/next of kin.

## Author Contributions

X-JX, Z-BL, HS, W-QX, and M-HW participated in the data collection. NZ performed the cytokine analysis. X-JX and Z-BL performed the data analysis. X-JX, Y-MT, and J-IH designed the work and performed the data interpretation. X-JX and Y-MT drafted the manuscript. J-IH revised the manuscript. All authors contributed to the article and approved the submitted version.

## Funding

This work was supported by grants from the National Natural Science Foundation of China (Nos. 81970122, 81770202), the Zhejiang Provincial Natural Science Foundation of China (No. LY19H080006), the Key Project of the Science and Technology of Zhejiang Province (2019C03032), and the Pediatric Leukemia Diagnostic and Therapeutic Technology Research Center of Zhejiang Province (No. JBZX-201904).

## Conflict of Interest

The authors declare that the research was conducted in the absence of any commercial or financial relationships that could be construed as a potential conflict of interest.

## Publisher’s Note

All claims expressed in this article are solely those of the authors and do not necessarily represent those of their affiliated organizations, or those of the publisher, the editors and the reviewers. Any product that may be evaluated in this article, or claim that may be made by its manufacturer, is not guaranteed or endorsed by the publisher.
