# Supplementary material for: Simple Evaluation of Clinical Situation and Subtypes of Pediatric Hemophagocytic Lymphohistiocytosis by Cytokine Patterns
Source: Front Immunol. 2022 Feb 28;13:850443. doi: 10.3389/fimmu.2022.850443 (PMC8918565; doi:10.3389/fimmu.2022.850443)
Supplement: Supplementary file 1 [file Table_1.docx]

**Supplementary Table 1. Pathogenic or likely pathogenic variants identified in patients with primary HLH**

| Patient No. | Sex | Age at onset | Affected siblings | Gene | Mutation | Effect | Zygosity | HSCT | Outcome |
| --- | --- | --- | --- | --- | --- | --- | --- | --- | --- |
| UPN10 | F | 6.2y | No | *PRF1* | c.757G>A | p.E253K | Heterozygous | No | Died |
|  |  |  |  |  | c.1061A>T | p.D354V | Heterozygous |  |  |
| UPN51 | M | 1.3y | No | *PRF1* | c.680T>A | p.V227E | Heterozygous | No | Died |
|  |  |  |  |  | c.-2C>A | Altered Splicing | Heterozygous |  |  |
| UPN94 | M | 1.7y | No | *PRF1* | c.1349C>T | p.T450M | Heterozygous | No | Died |
|  |  |  |  |  | c.983G>C | p.W328S | Heterozygous |  |  |
| UPN104 | F | 0.75y | No | *PRF1* | c.1066C>T | p.A356T | Heterozygous | No | Died |
|  |  |  |  |  | c.1090_1091delCT | p.l364Gfs*93 | Heterozygous |  |  |
| UPN16 | M | 3d | Yes | *UNC13D* | c.2295_2298 delGCAG | p.E765Dfs*27 | Heterozygous | No | Died |
|  |  |  |  |  | c.2296C>T | p.Q766* | Heterozygous |  |  |
| UPN53 | M | 4.1y | No | *UNC13D* | c.2986G>A | p.G996R | Heterozygous | No | Alive |
|  |  |  |  |  | c.118-307G>A | Regulatory | Heterozygous |  |  |
| UPN150 | F | 0.8y | No | *UNC13D* | c.2405T>C | p.L802P | Heterozygous | No | Alive |
|  |  |  |  |  | c.1591+1G>C | Altered Splicing | Heterozygous |  |  |
| UPN154 | M | 2.5y | No | *UNC13D* | c.2759A>C | p.Y920C | Heterozygous | No | Alive |
|  |  |  |  |  | c.2709+1G>A | Altered Splicing | Heterozygous |  |  |
| UPN168 | F | 0.3y | No | *UNC13D* | c.1055+1G>A | Altered Splicing | Heterozygous | Yes | Alive |
|  |  |  |  |  | c.614+1G>T | Altered Splicing | Heterozygous |  |  |
| UPN171 | M | 3.7y | No | *UNC13D* | c.1232G>C | p.R411Q | Heterozygous | No | Alive |
|  |  |  |  |  | c.2633A>G | p.Q878R | Heterozygous |  |  |
|  |  |  |  |  | c.1189G>A | p.A397T | Heterozygous |  |  |
| UPN195 | M | 54d | No | *UNC13D* | c.2625+2T>A | Altered Splicing | Heterozygous | Yes | Alive |
|  |  |  |  |  | c.1055+1G>A | Altered Splicing | Heterozygous |  |  |
| UPN227 | M | 6d | No | *UNC13D* | c.1055+1G>A | Altered Splicing | Heterozygous | No | Died |
|  |  |  |  |  | c.118-308C>T | Regulatory | Heterozygous |  |  |
| UPN56 | M | 58d | Yes | *STXBP2* | c.704G>T | p.R235L | Heterozygous | No | Died |
|  |  |  |  |  | c.37+5G>C | Altered Splicing | Heterozygous |  |  |
| UPN54* | F | 1.0y | Yes | *STXBP2* | c.497C>T | p.T166M | Heterozygous | Yes | Alive |
| UPN136* | M | 37d | Yes | *STXBP2* | c.497C>T | p.T166M | Heterozygous | Yes | Died |
| UPN248 | M | 37d | No | *RAB27A* | c.509_510delTA | p.I170Kfs*5 | Heterozygous | No | Died |
|  |  |  |  |  | c.148dupA | p.R50Kfs*34 | Heterozygous |  |  |
| UPN207 | F | 2.4y | No | *LYST* | c.8473G>T | p.G2825C | Heterozygous | No | Alive |
|  |  |  |  |  | c.369A>G | p.H123R | Heterozygous |  |  |
| UPN17 | M | 0.9y | No | *SH2D1A* | c.162C>G | p.Y54X | Hemizygous | No | Died |
| UPN18 | M | 3.2y | Yes | *SH2D1A* | c.191G>A | p.W64X | Hemizygous | No | Died |
| UPN32 | M | 0.9y | No | *SH2D1A* | c.163C>T | p.R55X | Hemizygous | No | Died |
| UPN59 | M | 5.9y | No | *SH2D1A* | c.7G>T | p.A3S | Hemizygous | No | Alive |
| UPN98 | M | 4.8y | No | *BIRC4* | c.910G>T | p.G304X | Hemizygous | Yes | Died |
| UPN194 | M | 7.7y | Yes | *SH2D1A* | c.201+1G>T | Altered Splicing | Hemizygous | No | Died |
| UPN203 | M | 4.7y | No | *BIRC4* | c.1196T>G | p.I399R | Hemizygous | No | Alive |
| UPN206 | M | 3.0y | No | *SH2D1A* | *SH2D1A* deletion |  | Hemizygous | No | Died |
| UPN237 | M | 1.9y | No | *SH2D1A* | c.137+5G>A | Altered Splicing | Hemizygous | No | Lost to follow-up |

*UPN54 and UPN136 are siblings.
